# 术前应用靶向药物治疗*ALK*阳性NSCLC患者的围手术期安全性分析

**DOI:** 10.3779/j.issn.1009-3419.2026.106.19

**Published:** 2026-06-20

**Authors:** Mengying FAN, Cunwei QIN, Yifan FANG, Hao FU, Liang DAI, Yongbo YANG, Zhen LIANG, Hongchao XIONG, Wanpu YAN, Keneng CHEN

**Affiliations:** ^1^100142 北京，北京市癌症与转化研究重点实验室，北京大学肿瘤医院胸外一科（范梦颖，方一凡，付浩，戴亮，杨永波，梁震，熊宏超，闫万璞，陈克能）; ^1^Key Laboratory of Carcinogenesis and Translational Research (Ministry of Education/Beijing), Department I of Thoracic Surgery, Peking University Cancer Hospital & Institute, Beijing 100142, China; ^2^277500 滕州，滕州市中心人民医院胸外科（秦存伟）; ^2^Department of Thoracic Surgery, Tengzhou Central People’s Hospital, Tengzhou 277500, China; ^3^100142 北京，国家分子肿瘤学重点实验室（陈克能）; ^3^State Key Laboratory of Molecular Oncology, Beijing 100142, China

**Keywords:** 肺肿瘤, 新辅助靶向治疗, 间变性淋巴瘤激酶, 安全性, Lung neoplasms, Neoadjuvant targeted therapy, Anaplastic lymphoma kinase, Safety

## Abstract

**背景与目的:**

局部晚期非小细胞肺癌（non-small cell lung cancer, NSCLC）的围手术期治疗一直是临床难点。虽然间变性淋巴瘤激酶-酪氨酸激酶抑制剂（anaplastic lymphoma kinase-tyrosine kinase inhibitors, ALK-TKIs）在*ALK*阳性NSCLC的术后辅助治疗中已确立重要地位，但其在术前新辅助治疗中的应用证据仍较匮乏。ALNEO等探索性研究提示术前ALK-TKIs治疗可带来比较理想的主要病理缓解（major pathological response, MPR）率，但围手术期安全性的临床报道依然有限。本研究旨在通过回顾性分析单中心数据评估术前ALK-TKIs治疗的围手术期安全性与可行性。

**方法:**

回顾性分析2021至2024年北京大学肿瘤医院胸外一科前瞻性数据库中记录的接受术前ALK-TKIs靶向治疗并完成手术切除的*ALK*阳性NSCLC患者资料，评估其近期疗效、手术相关指标、围手术期并发症及远期生存情况。

**结果:**

全组共11例患者，以女性并不吸烟患者为主（9/11），病理类型均为腺癌。治疗前临床分期为IIIA期7例、IIIB期3例、IV期寡转移1例。全组患者术前均接受ALK-TKIs治疗，其中4例联合化疗。客观缓解率（objective response rate, ORR）为81.8%。所有患者均顺利完成胸腔镜或机器人辅助手术，平均手术时间为（122.0±62.3）min，中位术中出血量74.0 mL（范围：5.0-300.0 mL）。MPR率为45.5%（5/11），病理完全缓解（pathological complete response, pCR）率为27.3%（3/11）。所有患者术后均接受了以靶向治疗为主的辅助治疗。中位随访30个月，全组无死亡病例，3例患者出现复发转移，其中1例为肠和肝转移，2例为脑转移。

**结论:**

在本小样本队列中*ALK*阳性的NSCLC患者接受术前ALK-TKIs治疗显示出较好的安全性和可行性。

## Abstract (en)

**Background and objective:**

Perioperative management of locally advanced non-small cell lung cancer (NSCLC) remains a significant clinical challenge. Although anaplastic lymphoma kinase-tyrosine kinase inhibitors (ALK-TKIs) have established a definitive role in the adjuvant setting for *ALK*-positive NSCLC, clinical evidence supporting their preoperative application remains scarce. Recent exploratory trials, such as the ALNEO study, indicated that preoperative ALK-TKIs therapy can achieve favorable major pathological response (MPR) rates; however, documented data regarding its perioperative safety remain limited. This study aims to evaluate the perioperative safety and feasibility of preoperative ALK-TKIs therapy through a retrospective, single-center analysis.

**Methods:**

A retrospective review was conducted using the data of *ALK*-positive NSCLC patients recorded in the prospective database of Department I of Thoracic Surgery at Peking University Cancer Hospital from 2021 to 2024, who underwent surgical resection following preoperative ALK-TKIs therapy. Short-term efficacy, surgical outcomes, perioperative complications, and long-term survival data were systematically evaluated.

**Results:**

Eleven patients were included, predominantly consisting of female non-smokers (9/11), and the pathological type for all patients was adenocarcinoma. Baseline clinical staging included stage IIIA (*n*=7), stage IIIB (*n*=3), and stage IV with oligometastasis (*n*=1). All patients received preoperative ALK-TKIs, with 4 receiving concurrent chemotherapy. The objective response rate (ORR) was 81.8%. All patients successfully underwent surgery via video- or robotic-assisted thoracoscopic surgery. The mean operative time was (122.0±62.3) min and the median intraoperative blood loss was 74.0 mL (range: 5.0-300.0 mL). The MPR and pathological complete response (pCR) rates were 45.5% (5/11) and 27.3% (3/11), respectively. All patients received postoperative adjuvant therapy, primarily targeted drugs. At a median follow-up of 30 months, no deaths occurred in the entire cohort; however, three patients experienced disease recurrence (one with intestinal and liver metastases, and two with brain metastases).

**Conclusion:**

Preoperative ALK-TKIs therapy demonstrates favorable safety and feasibility in this small-sample cohort of *ALK*-positive NSCLC patients.

肺癌仍是全球发病率与死亡率最高的恶性肿瘤之一，初诊即为局部晚期的患者，如可切除的N2/III期非小细胞肺癌（non-small cell lung cancer, NSCLC），是临床治疗上的难点^[1]^。对于此类患者，传统治疗策略强调多学科综合治疗，术前治疗是最受关注的一部分^[2]^，原因在于术前治疗（或新辅助治疗）可在手术前期控制微转移灶、缩小肿瘤体积、提高R0切除率，并为后续治疗策略提供“在体”疗效评估结果^[3]^。

随着精准治疗时代的到来，NSCLC的术前治疗日益强调以生物标志物为核心的个体化策略。程序性死亡配体1（programmed death-ligand 1, PD-L1）表达水平以及驱动基因如表皮生长因子受体（epidermal growth factor receptor, *EGFR*）、间变性淋巴瘤激酶（anaplastic lymphoma kinase, *ALK*）基因突变等，常用于甄别免疫治疗、化疗与靶向治疗的获益人群。对于*ALK*基因融合突变（*ALK*突变）的患者，术后辅助治疗领域目前推荐使用ALK-酪氨酸激酶抑制剂（tyrosine kinase inhibitors, TKIs）^[4,5]^；然而，目前支持ALK-TKIs用于术前治疗的证据相对匮乏，近年来仅有ALNEO^[6]^与LORIN^[7]^等探索性研究初步提示：将ALK-TKIs用于术前治疗能带来较为理想的影像与病理学的缓解，为这类人群的围手术期治疗提供了新的希望。

真实世界中在评估是否将ALK-TKIs用于术前治疗时，除了关注疾病缓解、肿瘤降期与R0切除等获益之外，还必须面对一个数据更少但同等重要的问题，即安全性，尤其是围手术期的安全，具体而言，如ALK-TKIs相关的不良反应及其是否导致剂量调整，治疗后组织水肿、纤维化及粘连对手术操作的影响，以及术前治疗是否会增加手术并发症、延长胸管留置及住院时间。这些关键的安全性问题均可能影响到临床决策。由于*ALK*基因突变概率较低，接受术前治疗者更为稀缺，因此目前关于安全性数据的报道仍然较少。

基于上述临床空白，本课题组开展了本项回顾性研究，旨在填补ALK-TKIs在术前治疗领域关于围手术期安全性的证据缺口，研究的假设是ALK-TKIs用于术前治疗并不会增加患者的不良事件（adverse events, AEs），不会影响后续的手术治疗。

## 1 资料与方法

### 1.1 临床资料

本研究回顾性分析了2021至2024年北京大学肿瘤医院胸外一科前瞻性数据库中连续性术前接受了ALK-TKIs治疗的NSCLC患者，对其围手术期安全性、近期疗效及远期生存情况进行分析。入组标准：（1）病理证实为NSCLC；（2）临床分期为III期及以上；（3）基因检测提示*ALK*突变阳性[免疫组化、荧光原位杂交（fluorescence *in situ* hybridization, FISH）或下一代测序（next-generation sequencing, NGS）]；（4）术前接受了ALK-TKIs靶向治疗者。排除标准：（1）未接受手术治疗；（2）术前未接受靶向治疗。本研究已获得北京大学肿瘤医院伦理委员会批准（No.2023KT86）。

### 1.2 分期方法及随访内容

在治疗开始前30天内进行标准的分期检查，包括胸部增强计算机断层扫描（computed tomography, CT）、正电子发射型计算机断层显像（positron emission tomography/CT, PET/CT）、头颅磁共振成像（magnetic resonance imaging, MRI）检查。对于不能进行核磁检查者，可采用头颅增强CT替代。对于未进行PET/CT检查者，需进行腹部超声（包括肝脏、肾上腺）或上腹部增强CT检查及全身骨扫描检查。对于CT或PET/CT检查提示肺门及纵隔可疑的淋巴结，推荐采用经支气管镜超声引导下针吸活检术（endobronchial ultrasound-guided transbronchial needle aspiration, EBUS-TBNA）或其他活检方式明确病理诊断。所采用的分期标准为美国癌症联合委员会（American Joint Committee on Cancer, AJCC）第8版分期^[8]^。

### 1.3 新辅助治疗适应证及方法

对于cN2的患者均进行新辅助治疗；对于cN1以及T2以上的N0患者，由包括肿瘤内科、肿瘤外科、放疗科、影像科在内进行多学科诊疗（multi-disciplinary treatment, MDT）团队讨论决定是否进行新辅助治疗。若患者联合新辅助化疗，每21天重复1次，总共进行2-4个周期，具体周期数由MDT团队决定。新辅助治疗方案中化疗方案为以铂类为基础的双药联合方案，根据患者的病理类型及研究者决定。包括：培美曲塞联合卡铂，培美曲塞500 mg/m^2^（第1天）；卡铂曲线下面积（area under the curve, AUC）为5（卡铂计算采用公式，根据身高、体重、性别、年龄、肌酐及AUC取值决定）（第1天），每21天为1个周期。靶向治疗药物包括阿来替尼、恩沙替尼、洛拉替尼。

### 1.4 新辅助治疗后再分期的方式

新辅助治疗后手术前30天内，采用胸部增强CT重新评估T和N分期（11例中有9例患者使用PET评估），若距离初次评估超过90天者会复查PET/CT及头颅增强MRI排除远处转移。对于新辅助治疗后仍怀疑N2或N3淋巴结阳性的患者，会重新活检明确病理诊断。评效采用实体瘤疗效评价标准（Response Evaluation Criteria in Solid Tumors, RECIST）1.1进行影像学评估^[9]^。

联合化疗者每3天复查1次血常规，每周1次复查肝肾功能；单纯TKIs靶向治疗者每月复查1次肝肾功能及血常规。围手术期治疗AEs按照不良事件常用术语标准（Common Terminology Criteria for Adverse Events, CTCAE）4.0^[10]^进行评估。

### 1.5 手术切除的适应证及方式

新辅助治疗后再分期为I-IIIA期的患者接受手术治疗，cN2患者由MDT团队讨论决定是否行手术切除。手术切除范围以肺叶切除为主，部分中心型病灶会接受联合肺叶切除、袖式肺叶切除或全肺切除术。淋巴结清扫范围多为系统性淋巴结清扫，至少包括3站N1和3站N2淋巴结（N2淋巴结要求包括7组淋巴结）。

### 1.6 病理评估方式

术后病理退缩程度评估根据国际肺癌研究协会（International Association for the Study of Lung Cancer, IASLC）的标准^[11]^，要求全部病灶连续切片，根据肿瘤、坏死、纤维化的区域计算瘤床，每个中心由两位以上经验丰富的病理科医生独立评估。

### 1.7 术后治疗的原则

术后辅助治疗与否以及辅助治疗的具体方案，由MDT团队根据当时的治疗指南讨论决定。

### 1.8 随访方法

手术后前2年内，每3个月复查血液学检查、胸部增强CT、腹部超声（包括肝脏、肾上腺），每6-12个月复查头颅增强MRI；手术后第3-5年，每6个月复查血液学检查、胸部增强CT、腹部超声（包括肝脏、肾上腺），每年复查头颅增强MRI；手术后5年以后，每年复查血液学检查、胸部增强CT、腹部超声（包括肝脏、肾上腺）、头颅增强MRI。对于不能完成核磁检查者，可采用头颅增强CT替代。

### 1.9 研究终点

本研究主要研究终点为围手术期安全性（包括药物及手术相关AEs）、手术操作难度，次要研究终点为新辅助治疗后的主要病理缓解（major pathological response, MPR）率、病理完全缓解（pathological complete response, pCR）率和远期生存。本文报告数据的截止日期为2026年1月4日。

### 1.10 统计学方法

本研究主要采用描述性统计分析，所有数据分析均使用R软件（for Windows, version 4.3.2）完成。连续变量根据其分布特征，符合正态分布时采用均数、标准差、范围描述；不符合正态分布时使用中位数、四分位距（interquartile range, IQR）或范围进行描述。分类变量采用例数和百分比（%）进行描述。生存分析采用*Kaplan-Meier*法计算生存率。

## 2 结果

### 2.1 本组患者的一般情况

本研究共纳入接受新辅助靶向治疗并手术的*ALK*阳性NSCLC患者11例。其中男性2例（18.2%），女性9例（81.8%），男女比例为1:4.5。平均年龄51.5岁（范围：38-65岁）。病理类型均为腺癌。临床分期IIIA期7例，IIIB期3例，IV期1例。病理确认N分期者5例（其中N2者4例、N3者1例）。患者一般资料及具体临床分期见表1。

**表 1:** 患者的一般资料及临床特征（*n*=11）

Characteristics		Data
Age, yrs	Mean	51.5
	Range	38.1-65.8
Sex	Male	2 (18.2%)
	Female	9 (81.8%)
ECOG performance status	0	11 (100.0%)
Smoking status	Ever	2 (18.2%)
	Never	9 (81.8%)
Tumor location	Right upper lobe	3 (27.3%)
	Right middle lobe	1 (9.1%)
	Right lower lobe	4 (36.4%)
	Left upper lobe	3 (27.3%)
Histology	Adenocarcinoma	11 (100.0%)
ALK detection method	Ventana	6 (54.5%)
	PCR	3 (27.3%)
	NGS	2 (18.2%)
Clinical T stage	cT1	5 (45.5%)
	cT2	3 (27.3%)
	cT3	1 (9.1%)
	cT4	2 (18.2%)
Clinical N stage	cN0	2 (18.2%)
	cN1	0 (0.0%)
	cN2	8 (72.7%)
	cN3	1 (9.1%)
Clinical stage	cIIIA	7 (63.6%)
	cIIIB	3 (27.3%)
	cIV	1 (9.1%)
yp stage	yp0	3 (27.3%)
	ypIA	3 (27.3%)
	ypIIB	1 (9.1%)
	ypIIIA	3 (27.3%)
	ypIIIB	1 (9.1%)
Adjuvant therapy	Yes	11 (100.0%)

ECOG: Eastern Cooperative Oncology Group; ALK: anaplastic lymphoma kinase; PCR: polymerase chain reaction; NGS: next-generation sequencing; yp: pathological stage after neoadjuvant therapy.

### 2.2 术前治疗方案、AEs及疗效

全组患者均接受术前靶向治疗，其中4例患者接受化疗联合靶向治疗，7例患者接受单纯靶向治疗。术前化疗周期为2-4个周期，化疗方案均为培美曲塞联合卡铂。靶向药物包括阿来替尼、恩沙替尼及洛拉替尼（表2），初始剂量均参考晚期适应证获批剂量，术前靶向药物治疗时长为2-20个月，中位靶向时间为5.0个月（2.0-20.0 个月）。

**表 2:** 患者新辅助治疗和手术情况（*n*=11）

Characteristics		Data
Chemotherapy	Yes	4 (36.4%)
	No	7 (63.6%)
TKIs	Alectinib	4 (36.4%)
	Ensartinib	6 (54.5%)
	Lorlatinib	1 (9.1%)
Radiologic response	PR	9 (81.8%)
	SD	1 (9.1%)
	PD	1 (9.1%)
Downstaging	Yes	8 (72.7%)
	No	3 (27.3%)
Surgical approach	VATS	10 (90.9%)
	RATS	1 (9.1%)
Extent of resection	Lobectomy	9 (81.8%)
	Wedge resection	2 (18.2%)
Duration from clinic to surgery, mon, median (IQR)		5.0 (2.5-13.1)
Operation time, min, mean (SD)		122.0 (62.3)
Blood loss, mL, median (range)		74.0 (5.0-300.0)

TKIs: tyrosine kinase inhibitors; PR: partial response; SD: stable disease; PD: progressive disease; VATS: video-assisted thoracoscopic surgery; RATS: robot-assisted thoracoscopic surgery; IQR: interquartile range; SD: standard deviation.

术前治疗期间发生3级以上AEs者1例（水肿，占9.1%）；其他治疗相关副作用多为1-2级，以皮疹、转氨酶升高多见，未观察到间质性肺炎，无治疗相关死亡发生，未出现因AEs导致的减量或停药（表3）。

**表 3:** 术前和术后AEs（*n*=11）

Item	Preoperative AEs			Postoperative AEs
	Grade 1-2	Grade 3		Grade 1-2
Edema	0	1		0
Rash	4	0		4
Elevated transaminases	4	0		4
AEs leading to dose reduction or discontinuation	0	0		0
AEs leading to surgical delay or cancellation	0	0		-

No grade≥3 AEs occurred during the postoperative phase. AEs: adverse events.

根据RECIST标准，影像学评估客观缓解率（objective response rate, ORR）为81.8%（9/11），其中部分缓解（partial response, PR）为9例，疾病稳定（stable disease, SD）为1例，疾病进展（progressive disease, PD）（原发灶PR、淋巴结PD）为1例。治疗后降期者8例（72.7%），其中T降期者8例，N降期者5例，N2降期者3例（37.5%, 3/8）。

### 2.3 手术情况和围手术期安全性

全组患者中8例术前未停靶向药物，1例患者术前4周停药，2例患者术前6-7周停药；没有出现因药物相关AEs而推迟手术者。患者均接受以根治性切除为目的的手术治疗，10例接受电视辅助胸腔镜手术（video-assisted thoracoscopic surgery, VATS），1例行机器人辅助胸腔镜手术（robot-assisted thoracoscopic surgery, RATS），无术中中转开胸者。9例患者行肺叶切除术，并进行系统性淋巴结清扫；2例患者行肺楔形切除术（1例为IV期寡转移者，1例为原发灶PR但淋巴结PD者，行纵隔淋巴结清扫联合原发灶切除）。全组患者的平均手术时间为（122.0±62.3）min，中位出血量为74.0 mL（范围：5.0-300.0 mL）。术中记录了粘连、斑痕者5例，1例患者术中记录组织水肿严重，与靶向治疗相关。9例患者行系统性淋巴结清扫，1例患者行淋巴结采样，1例患者（IV期孤立骨转移）未行淋巴结清扫，其中3例淋巴结与周围组织粘连紧密，清扫困难，与靶向治疗反应有关。无患者术中及术后输血，无术后入重症监护室（intensive care unit, ICU）者。无血管损伤、无支气管成形/血管成形。TKIs联合化疗组患者相比TKIs单药组患者，在上述指标中未见明显差异。

术后平均拔除胸管时间为2天（范围：1-4天）。术后平均住院日4.33天（范围：2-8天）。全组患者无二次入院及二次引流者。1例患者因术后气管水肿，咳痰困难而行支气管镜吸痰，其余患者无围手术期相关并发症，全组患者无治疗相关死亡。

### 2.4 病理分析结果

实现R0切除者11例（100.0%）。手术后评估MPR者5例（45.5%），pCR者3例（27.3%）。术后病理分期yp0期者3例（27.3%），ypIA期者3例（27.3%），ypIIB期者1例（9.1%），ypIIIA期者3例（27.3%），ypIIIB期者1例（9.1%），具体信息见图1和图2。实现N分期降期者6例：N3到N0者1例，N2到N0者4例，N2到N1者1例。

**图 1:**
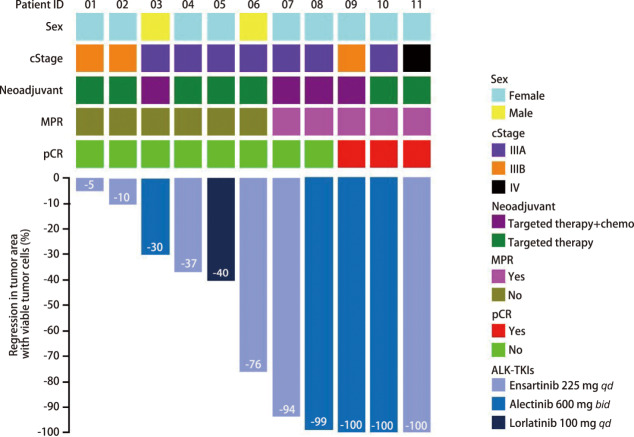
新辅助治疗疗效瀑布图

**图 2:**
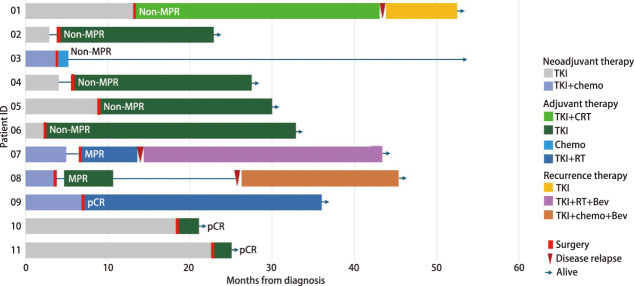
新辅助治疗者游泳图

### 2.5 术后治疗情况及远期预后

所有患者均接受术后辅助治疗。1例患者术后复测FISH及NGS，判定*ALK*阴性，遂行术后辅助化疗2个周期；其余患者接受靶向辅助治疗，药物均为第二代ALK-TKIs，辅助靶向治疗时长（6个月-2年+）。1例术前cN3患者行颈部放疗，2例yp多站N2者行术后放疗。术后治疗过程中无3级以上AEs记录。

本组患者中位随访时间为30.0个月（范围：18.5-52.8个月），术后1年无病生存（disease-free survival, DFS）率为85.7%。目前，11例患者中有3例（27.3%）患者出现复发转移，均为远处转移。患者08初始分期为cIIIA期，术前接受阿来替尼联合化疗，达到MPR，术后1个月时开始接受阿来替尼单药辅助治疗半年，停药1年后出现肠转移、肝转移，再次行原方案靶向治疗有效；患者01初始分期为cIIIB期，术前接受恩沙替尼，非MPR，术后接受恩沙替尼联合放化疗，于术后30.1个月复发。患者07初始分期为cIIIA期，术前接受恩沙替尼联合化疗，达到MPR，术后接受恩沙替尼联合放疗，于术后7.9个月复发。患者01和07均在辅助靶向治疗期间出现脑转移，更换第三代ALK-TKIs并行脑立体定向放疗有效。11例患者中无患者因PD导致死亡。

## 3 讨论

*ALK*基因融合是NSCLC重要的驱动基因之一，其在NSCLC人群中的比例为5%-8%^[12]^，相较于其他驱动基因，*ALK*融合的患者多为年轻、女性、不吸烟、腺癌患者，且恶性程度高，较早出现淋巴结转移及脑转移^[4]^。临床上确诊的*ALK*突变的III期以上NSCLC患者比例远高于驱动基因野生型患者，因此术前治疗对于该类患者的治疗意义重大。然而，目前大多数探讨III期患者的术前新辅助治疗的临床研究均将*ALK*突变患者排除在外（如CheckMate 816^[13]^、RATIONALE-315^[14]^等研究）。本研究所能参考数据仅有如下几项：KEYNOTE-671^[15]^、AEGEAN研究^[16]^，虽入组开始未排除*ALK*阳性患者，但因例数较少，在后续分析中并未单独报道这部分患者的数据情况。此外，Zhang等^[17]^报道了应用克唑替尼治疗N2患者的结果，其pCR率为18.2%（2/11）。ALNEO试验^[6]^共计入组33例*ALK*阳性患者，术前行8周阿来替尼治疗后行手术治疗，其手术率为85%（28/33），R0切除率为86%（24/28）。MPR率为42%，pCR率为12%。LORIN研究^[7]^入组III期*ALK*阳性患者行洛拉替尼新辅助治疗3个周期，该研究报道的13例患者中，11例接受了手术治疗，其MPR率高达76.9%，pCR率为23.1%。另外，非随机对照研究^[18]^对比了阿来替尼和克唑替尼用于III期NSCLC患者术前治疗的效果，两组的MPR率分别为64.7%与46.2%。此外，意大利的多中心回顾性研究^[19]^也表明阿来替尼对于局部晚期或转移性*ALK*阳性NSCLC患者，其pCR率可达50%。根据以上研究数据及本研究结果，本课题组认为*ALK*突变的III期可切除NSCLC患者的术前靶向治疗的效果优于单纯化疗或者化疗联合免疫治疗，此类患者的术前治疗方案应该优先考虑ALK-TKIs。

在药物不良反应方面，目前上市的ALK-TKIs的常见AEs为便秘、肝功能异常、贫血、疲劳、血胆红素升高、高脂血症等。多项研究报道了ALK-TKIs在术前治疗期间的不良反应。例如，ALNEO研究^[6]^报道新辅助治疗3级AEs为转氨酶增高、腹泻及肌酸磷酸激酶增高（各1例，3%），2例患者因AEs导致剂量减少。LORIN研究^[7]^中30.8%的患者在新辅助阶段出现3级及以上高胆固醇血症/高甘油三酯血症，38.5%患者因AEs导致剂量减少或暂时停药。既往研究及本研究无因AEs导致推迟手术，结合本研究的数据课题组认为术前使用ALK-TKIs的安全性是可接受的。

关于术前治疗另外一个需要考虑的问题就是其对于后续手术的影响。在这方面首先要评估其是否会延误甚至使患者错失手术时机。ALNEO研究^[6]^中未行手术治疗的5例患者中，1例为PD，2例为临床决定不适合手术，2例为患者拒绝手术。而在LORIN研究^[7]^中，2例初治不可切除患者因评估SD未行手术治疗。此外，本课题组仍需评估其是否会增加手术难度。LORIN研究^[7]^中1例患者中转开胸，术中出血量最高800 mL（中位出血量60 mL）。意大利多中心回顾性分析^[19]^中，10例接受手术的患者60%为开胸手术，7例手术出现术中困难，主要表现为血管周围纤维化或纵隔淋巴结清扫困难。本研究中所有患者的中位药物暴露时间为5个月，而且有72.7%的患者术前并未停药。即便如此，11例患者均成功接受了腔镜手术，无术中大出血及ICU入住，本组患者术中出血、围手术期并发症均与已有研究相似^[7]^。因此，本课题组认为目前尚无数据表明术前使用ALK-TKIs会增加手术操作的难度和降低围手术期的安全性。

基于ALINA研究^[20]^，II-III期*ALK*阳性患者术后接受阿来替尼辅助靶向治疗可降低复发或死亡风险76%（HR=0.24），*ALK*突变患者的术后治疗推荐选择ALK-TKIs。但是，目前尚无研究探讨接受了术前TKIs治疗的NSCLC患者术后治疗该如何选择。ALNEO研究^[6]^术后行2年辅助靶向治疗，有18%患者出现PD（*n*=6）。LORIN研究^[7]^采用分子残留病灶指导的术后辅助治疗，9例患者未接受术后辅助治疗，其中1例患者复发。本研究中除1例*ALK*转阴患者接受辅助化疗外，其余所有患者均接受第二代TKIs药物靶向辅助治疗，其中3例患者复发，2例更换第三代药物有效，1例继续服用第二代药物有效。在安全性方面ALNEO研究^[6]^报道了在辅助治疗阶段AEs的数据，11.5%（3/26）出现3级AEs，1例为转氨酶增高，1例为中性粒细胞减少，1例为腹痛。本研究术后治疗的AEs多为1-2级，与既往研究相似，无新发信号，无3级以上AEs。综合考虑术后使用ALK-TKIs的疗效及安全性，本课题组认为针对接受了术前TKIs治疗的III期NSCLC患者，在完成手术切除后仍应该考虑继续使用TKIs治疗。

本研究作为一项回顾性研究具有如下局限性：（1）由于*ALK*突变的发生率较低，且在治疗前就确诊*ALK*突变的患者更为少见，导致本研究样本量较小，所以研究结果均有待于后续研究的验证，只为临床实践提供参考；（2）本研究未能前瞻性地收集研究时间段内所有接受ALK-TKIs治疗的III期NSCLC患者数据，所以对于术前ALK-TKIs是否会延误手术治疗时机的分析结果可能存在偏倚；（3）本研究并未前瞻性收集关于手术操作难度的数据，部分数据来自术者的主观判断，也可能会对研究的分析结果有负面的影响；（4）本研究并未设同期对照组，新辅助治疗也部分联合化疗，且治疗时间、术前停药期跨度大，结论需谨慎外推。

本研究的结果显示*ALK*突变的NSCLC患者接受术前ALK-TKIs治疗并不增加手术相关并发症，这提示我们临床实践需要一项前瞻性、大样本的III期随机对照研究来探索*ALK*突变III期NSCLC患者术前治疗的最佳治疗模式。

## References

[1] Bray F, Laversanne M, Sung H, et al. Global cancer statistics 2022: GLOBOCAN estimates of incidence and mortality worldwide for 36 cancers in 185 countries. CA Cancer J Clin, 2024, 74(3): 229-263. doi: 10.3322/caac.21834 38572751

[2] Oncology Society of Chinese Medical Association, Chinese Medical Association Publishing House. Chinese Medical Association guideline for clinical diagnosis and treatment of lung cancer (2023 edition). Zhonghua Yixue Zazhi, 2023, 103(27): 2037-2074. 37455124 10.3760/cma.j.cn112137-20230510-00767

[3] Muthusamy B, Patil PD, Pennell NA. Perioperative systemic therapy for resectable non-small cell lung cancer. J Natl Compr Canc Netw, 2022, 20(8): 953-961. doi: 10.6004/jnccn.2022.7021 35948038

[4] National Comprehensive Cancer Network. NCCN Clinical Practice Guidelines in Oncology (NCCN Guidelines®): Non-Small Cell Lung Cancer (Version 5. 2026). https://www.nccn.org/professionals/physician_gls/pdf/nscl.pdf.AccessedMarch22https://www.nccn.org/professionals/physician_gls/pdf/nscl.pdf.AccessedMarch22, 2026

[5] Guidelines Working Committee of the Chinese Society of Clinical Oncology ed. CSCO guidelines for diagnosis and treatment of non-small cell lung cancer 2025. Beijing: People's Medical Publishing House, 2025.

[6] Leonetti A, Boni L, Gnetti L, et al. Alectinib as neoadjuvant treatment in potentially resectable stage III *ALK*-positive NSCLC: Final analysis of ALNEO phase II trial (GOIRC-01-2020-ML42316). J Clin Oncol, 2025, 43(16_suppl): 8015. doi: 10.1200/JCO.2025.43.16_suppl.8015

[7] Zhong WZ, Zhang C, Yang Y, et al. P3.08F.01 Interim analysis of a phase 2 prospective trial of induction Lorlatinib in locally advanced *ALK*-positive NSCLC (LORIN). J Thorac Oncol, 2024, 19(10): S337-S338. doi: 10.1016/j.jtho.2024.09.607

[8] Amin MB, Edge SB, Greene FL, et al., eds. AJCC cancer staging manual. 8^th^ ed. New York: Springer, 2017.

[9] Eisenhauer EA, Therasse P, Bogaerts J, et al. New response evaluation criteria in solid tumours: Revised RECIST guideline (version 1.1). Eur J Cancer, 2009, 45(2): 228-247. doi: 10.1016/j.ejca.2008.10.026 19097774

[10] U.S. Department of Health and Human Services. Common Terminology Criteria for Adverse Events (CTCAE) Version 4.0. https://evs.nci.nih.gov/ftp1/CTCAE/CTCAE_4.03/Archive/CTCAE_4.0_2009-05-29_QuickReference_8.5x11.pdfhttps://evs.nci.nih.gov/ftp1/CTCAE/CTCAE_4.03/Archive/CTCAE_4.0_2009-05-29_QuickReference_8.5x11.pdf.Accessed March 22, 2026

[11] Travis WD, Dacic S, Wistuba I, et al. IASLC multidisciplinary recommendations for pathologic assessment of lung cancer resection specimens after neoadjuvant therapy. J Thorac Oncol, 2020, 15(5): 709-740. doi: 10.1016/j.jtho.2020.01.005 32004713 PMC8173999

[12] Committee of Tumor Pathology of Chinese Anti-Cancer Association, Lung Cancer Expert Committee of Oncology Branch of Chinese Medical Association, National Center for Quality Control in Pathology. Chinese expert consensus on the clinical practice of fusion gene testing in non-small cell lung cancer (2023 edition). Zhonghua Binglixue Zazhi, 2023, 52(6): 565-573.

[13] Forde PM, Spicer J, Lu S, et al. Neoadjuvant Nivolumab plus chemotherapy in resectable lung cancer. N Engl J Med, 2022, 386(21): 1973-1985. doi: 10.1056/NEJMoa2202170 35403841 PMC9844511

[14] Yue D, Wang W, Liu H, et al. Perioperative Tislelizumab plus neoadjuvant chemotherapy for patients with resectable non-small-cell lung cancer (RATIONALE-315): An interim analysis of a randomised clinical trial. Lancet Respir Med, 2025, 13(2): 119-129. doi: 10.1016/S2213-2600(24)00269-8 39581197

[15] Wakelee H, Liberman M, Kato T, et al. Perioperative Pembrolizumab for early-stage non-small cell lung cancer. N Engl J Med, 2023, 389(6): 491-503. doi: 10.1056/NEJMoa2302983 37272513 PMC11074923

[16] Heymach JV, Harpole D, Mitsudomi T, et al. Perioperative Durvalumab for resectable non-small cell lung cancer. N Engl J Med, 2023, 389(18): 1672-1684. doi: 10.1056/NEJMoa2304875 37870974

[17] Zhang C, Li SL, Nie Q, et al. Neoadjuvant Crizotinib in resectable locally advanced non-small cell lung cancer with *ALK* rearrangement. J Thorac Oncol, 2019, 14(4): 726-731. doi: 10.1016/j.jtho.2018.10.161 30408570

[18] Zhang C, Liu S, Jiang B, et al. Induction Alectinib or Crizotinib for stage III NSCLC harboring *ALK* fusion: A study with 4-year follow-up. Chin Med J (Engl), 2026, 139(9): 1357-1366. doi: 10.1097/CM9.0000000000003798 41139663 PMC13120571

[19] Lococo F, Cancellieri A, Chiappetta M, et al. Salvage surgery after first-line Alectinib for locally-advanced/metastatic *ALK*-rearranged NSCLC: Pathological response and perioperative results. Clin Lung Cancer, 2023, 24(5): 467-473. doi: 10.1016/j.cllc.2023.03.008 37061413

[20] Wu YL, Dziadziuszko R, Ahn JS, et al. Alectinib in resected *ALK*-positive non-small cell lung cancer. N Engl J Med, 2024, 390(14): 1265-1276. doi: 10.1056/NEJMoa2310532 38598794

